# Prioritizing population oral health through public policy in Australia: the Victorian experience

**DOI:** 10.1093/heapro/daad086

**Published:** 2023-08-09

**Authors:** Tan Minh Nguyen, Clare Lin, Anil Raichur, Amy Patterson, Martin Hall, Rosemary Aldrich, Suzanne Robinson

**Affiliations:** Oral Health Economics Research Stream, Deakin Health Economics, Institute for Health Transformation, Faculty of Health, Deakin University, Melbourne, Victoria, Australia; Dental Health Services Victoria, Carlton, Victoria, Australia; Community Based Health Services Policy & Improvement, Commisioning and System Improvement, Victorian Department of Health, State Government of Victoria, Melbourne, Victoria, Australia; Dental Health Services Victoria, Carlton, Victoria, Australia; Dental Health Services Victoria, Carlton, Victoria, Australia; Grampians Public Health Unit, Ballarat, Victoria, Australia; Oral Health Economics Research Stream, Deakin Health Economics, Institute for Health Transformation, Faculty of Health, Deakin University, Melbourne, Victoria, Australia

**Keywords:** health policy, public health practice, health promotion, childhood obesity, non-communicable diseases

## Abstract

Dental caries, a non-communicable disease, is one of the most prevalent diseases globally and share common modifiable risk factors with obesity such as excess sugar intake. However, prioritization by governments to improve population oral health has been limited and is typically excluded from the discourse of public health policy development. Therefore, interventions that target dental caries can have other co-benefits including obesity prevention. In Victoria, Australia, local government authorities have a regulatory requirement to develop their Municipal Health and Wellbeing Plans. The aim of this paper is to identify whether prioritization for oral health by local government authorities in Victoria has changed through the subsequent renewal of the Victorian Public Health and Wellbeing Plans 2011–2015 and 2019–2023. Three desktop audits for all publicly available Municipal Health and Wellbeing Plans by local government authorities in Victoria were conducted between 2014 and 2022. Key terms related to oral health was searched within these policy documents and categorized into six indicators: (i) included oral health as a priority, (ii) linked healthy eating and oral health, (iii) supported the Achievement Program, (iv) included the Smiles 4 Miles program, (v) advocated for fluoridated drinking water, and (vi) included other strategies related to oral health. Overall, there was statistically significant reduction in five of the six indicators, with the exception for prioritization of other strategies related to oral health such as targeting excess sugar intake and smoking. A multi-sectoral approach, that includes oral health would be advantageous to address the growing burden of non-communicable diseases.

**Contribution to Health Promotion**

- A focus on oral health within public health planning is often implied through a common risk factor approach to addressing non-communicable diseases (NCDs).
- Emphasis in targeting oral health has potential co-benefits to preventing NCDs such as obesity from a life course approach.
- Embedding oral health as an explicit area of prioritization needs to be advocated by public health practitioners and health professionals in population health policy development.

## INTRODUCTION

Non-communicable diseases (NCDs) are one of the leading causes of mortality, accounting for 74% of all deaths globally (World Health Organization, 2022). In Australia, the release of the National Preventive Health Strategy 2021–30 reported 87% of deaths were due to chronic conditions (Department of Health and Aged Care, 2021). Oral diseases such as periodontitis have links with other NCDs including cardiovascular disease, diabetes mellitus, and chronic respiratory disease (Jin *et al.*, 2016) and affects over 3.5 billion people (Bernabe *et al*., 2020). (GBD 2017 Oral Disorders Collaborators *et al.*, 2020). Although a focus on prevention and using a systems approach is emphasized to address the social determinants of health, the impact of poor oral health is given little attention.

Dental caries as an NCD (Pitts *et al.*, 2021), remains a significant public health issue and is the most common cause of potentially preventable hospitalizations (PPHs) for oral conditions (Rogers *et al.*, 2018). In Australia, 40% of PPHs due to dental conditions were among children aged 0–14 years old (Australian Institute of Health and Welfare, 2023). Children living in poverty are especially affected by poor oral health, with impacts on speech development (Nadelman *et al.*, 2020) school attendance (Jackson *et al.*, 2011), and self-esteem and social success in adolescents (Guarnizo-Herreño and Wehby, 2012).

Australia’s National Oral Health Plan 2015–2024 reported dental caries among children had rapidly declined from mid-1970s to the mid-1990s, likely attributable to the expansion of community water fluoridation (COAG Health Council, 2015). Although the development and progression of dental caries is multi-factorial, it is diet-mediated and the role of excess free sugar intake remains the single most common cause (Sheiham and James, 2015). In particular, sugar sweetened beverages (SSBs), have been shown to be the greatest risk factor for children (Armfield *et al.*, 2013; Gibbs *et al.*, 2016). There is also emerging evidence that higher frequencies of SSBs intake are implicated with increased risk for and periodontal disease among young adults aged 18–25 years (Lula *et al.*, 2014; Gupta *et al.*, 2018).

The World Health Organization guidelines on free sugar intake recommend it should not exceed more than 10% of the total energy intake, with greater health benefits, including for dental caries prevention, with further reductions below 5% (World Health Organization, 2015). More than half of the Australian population exceed the World Health Organization recommendations on free sugar intake (Lei *et al.*, 2016). The Australian Dietary Guidelines includes specific advice to ‘Limit intake of foods and drinks containing added sugars such as confectionary, sugar-sweetened soft drinks and cordials, fruit drinks, vitamin waters, energy and sports drinks’ as part of the overarching guideline to ‘Limit intake of foods containing saturated fat, added salt, added sugars and alcohol’ (National Health and Medical Research Council, 2013).

Although causal relationships remain unclear, obesity and dental caries share a common modifiable risk factor (Chi *et al.*, 2017), namely excess sugar intake (Ravaghi *et al.*, 2020). Similarly, there are associations between overweight and obesity with periodontal health (Keller *et al.*, 2015). Studies among Australian children and adults have shown strong associations between overweight and obesity and dental caries (Barrington *et al.*, 2019; Ha *et al.*, 2022). From a life course perspective, the population prevalence of children affected by dental caries is significantly higher among children considered to be overweight or obese (Do and Spencer, 2016; Huse *et al.*, 2018; Graesser *et al.*, 2022).

Among adults, other NCDs such as cardiovascular disease (Sanz *et al.*, 2020), dementia (Xiangqun *et al.*, 2019), diabetes (Nascimento *et al.*, 2018) and obesity (Keller *et al.*, 2015) are implicated with poor periodontal health. These NCDs present later in the life course, which indicates that prevention, early detection and management for good oral health among children and adolescents is an important societal investment.

**Fig. 1:**
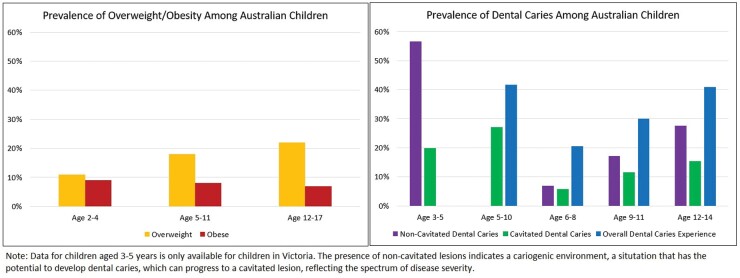
Prevalence of overweight/obesity, dental caries, and overall dental caries experience among Australian children (Do and Spencer, 2016; Huse *et al.*, 2018; Graesser *et al.*, 2022).

A comparison between overweight, obesity, dental caries and overall dental caries experience among Australian children is shown in Figure 1. It is important to note that the data should be interpreted with caution because dental caries prevalence is reported according to the deciduous and permanent dentition. Permanent teeth generally emerge from age 6 years, which potentially masks the existing modifiable ‘high’ risk factors that may continue to persist from early childhood, including excess sugar intake. Therefore, poor oral health among children, particularly regarding the prevalence and incidence of dental caries, could be viewed as an important indication of risk for overweight and obesity as both outcomes share common causal factors in relation to poverty and diet.

More than half of Victorian preschool children (56.6%) were diagnosed with early signs of non-cavitated dental caries (Graesser *et al.*, 2022) and almost half (43%) of all Victorian children aged 5–10 years have overall dental caries experience (dental caries and/or dental caries managed by having the tooth filled or extracted) (Do and Spencer, 2016). However, almost half (42.7%) of Australian children had never visited a dental practitioner before age 6 years, and this was relatively consistent across the age cohorts between 5 and 14 years (Do and Spencer, 2016). The presence of non-cavitated dental caries is generally difficult to diagnose without a clinical examination but indicates an individual’s immediate nutrition imbalance and higher risk towards poor oral health.

Utilization of the Child Dental Benefits Scheme, an Australian federal dental program for children aged 0–17 years, has also shown limited reach to only about one third of eligible children (Nguyen *et al.*, 2021). While access to dental services is important, broader factors such as socioeconomic influences are the biggest drivers in health status. Healthcare is estimated to be responsible for less than 25% of health status (Marmot and Allen, 2014). Therefore, a systems thinking approach is necessary to address complex public health issues (Khalil and Lakhani, 2022), such as obesity and oral health.

In Victoria 79 local government authorities have governance responsibilities for local governments areas (LGAs). This includes the development of a Municipal Public Health and Wellbeing Plan (MPHWP) every 4 years, which is a regulatory requirement under the *Public Health and Wellbeing Act 2008* (Victorian Department of Health, 2022a). In 2011, the first state-wide *Victorian Public Health and Wellbeing Plan (VPHWP) 2011–2015* was released, which provided high-level direction for a preventive and public health approach for promoting and maintaining the health and wellbeing of all Victorians (Department of Health. Victoria, 2011). Within this plan, oral health was explicitly considered a health priority. The MPHWPs are developed following the release of the state-wide VPHWPs.

Subsequently, the VPHWPs for 2015–2019 and 2019–2023 were developed and published. While oral health was not listed as a specific priority in either plan (Victorian Department of Health, 2015, 2019), risk factors common to oral health and other NCDs such as increasing healthy eating and reducing tobacco related harm were included as priorities. Other oral health policies were also developed, including the *Action Plan for Oral Health Promotion 2013–2017* (Victorian Department of Health, 2013), and the *Victorian Action Plan to Prevent Oral Disease 2020–2030*, which includes ‘Priority 2: Promote health Environments’ (Victorian Department of Health, 2020). In addition, the Victorian Department of Health and Dental Health Services Victoria (DHSV) developed oral health profiles and the local government action guide to assist LGAs to include oral health as a priority when developing their MPHWPs (Dental Health Services Victoria, 2020a).

There are three key Victorian state-wide health promotion programs, which are enablers for oral health: the Achievement Program, the Smiles 4 Miles program and the Healthy Eating Advisory Service. The Achievement Program is a Victorian state-wide initiative, which supports early childhood services, schools and workplaces to create healthy supportive environments, and include a priority for healthy eating and oral health (Cancer Council Victoria, 2018). Similarly, the Smiles 4 Miles program is specifically tailored to early childhood services, and centres around the promoting oral health messages of Eat Well, Drink Well, and Clean Well (Dental Health Services Victoria, 2016). The Healthy Eating Advisory Service provides free tailored support for organizations to provide healthy foods and drinks, and menus, including support to remove or reduce sugary drinks (Healthy Eating Advisory Service, 2022).

Between 2014 and 2022 three desktop audits were performed by the Victorian Department of Health and DHSV, to understand whether oral health was prioritized within local government authority MPHWPs, which were developed after the publication of the VPHWPs. The aim of this paper is to understand if oral health has been prioritized within the MPHWPs developed by Victorian local government authorities, and to compare changes between 2014 and 2022. This work informs future directions for public health policy development to promote oral health in Victoria through systems thinking.

## METHODS

This study evaluated the trends for prioritizing oral health from the three desktop audits for all publicly available local government authority MPHWPs. Pre-determined key terms related to oral health was searched within these policy documents, and categorized into six indicators,

- Included oral health as a priority (oral, dental, tooth, decay, caries).
- Linked healthy eating and oral health (sugar, sweet AND oral, dental, tooth, decay).
- Supported the Achievement Program (achievement).
- Included the Smiles 4 Miles program (smiles, s4m).
- Advocated for fluoridated drinking water (water, fluoride).
- Included other strategies related to oral health (sugar, smoking, diabetes etc.) (sugar, smoking, food, tobacco, diabetes, obesity).

For each indicator, relevant key terms found within the MPHWPs is given a count of 1, and its absence being 0. Each desktop audit summarized the proportion of local government authorities referencing the key terms for each indicator, and the results were published in a report for internal purposes (refer to Supplementary File). Data were recorded using Microsoft Excel 365 (Microsoft Corporation). Where there is a positive increase in the indicator, this is regarded as favourable, while a decrease is considered unfavourable.

**Fig. 2:**
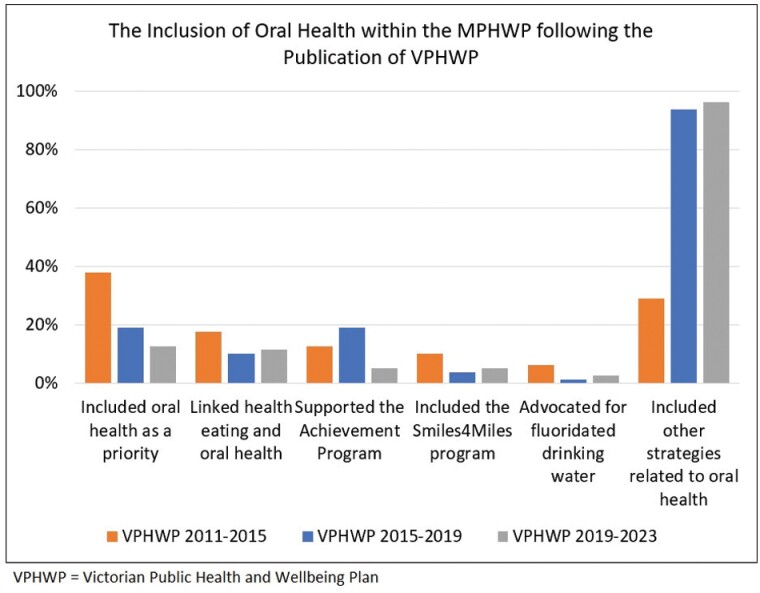
Trends of the indicators according to oral health key terms included in the MPHWPs following the publication of the VPHWPs.

The outcomes of the three desktop audits are presented in Table 1 and Figure 2. Mean-comparison tests of paired data (*t*-tests) were performed to determine whether there were any significant differences between the desktop audits. Data analysis was performed using Stata IC Version 12 (StatacorpTM). This study does not require ethics approval and conducted according to the Declaration of Helsinki.

**Table 1:** The proportion of MPHWPs reporting oral health key terms according to the indicators following the publication of the VPHWPs

Indicator	VPHWP 2011–2015 *N* = 79 (%)	VPHWP 2015–2019 *N* = 79 (%)	VPHWP 2019–2023 *N* = 79 (%)
Included oral health as a priority (%)	30 (38%)	15 (19%)	10 (13%)
Linked health eating and oral health (%)	14 (18%)	8 (10%)	9 (11%)
Supported the Achievement Program (%)	20 (25%)	15 (19%)	4 (5%)
Included the Smiles 4 Miles program (%)	8 (10%)	3 (4%)	4 (5%)
Advocated for fluoridated drinking water (%)	5 (6%)	1 (1%)	2 (3%)
Included other strategies related to oral health (sugar, smoking, diabetes etc.) (%)	29 (37%)	74 (94%)	76 (96%)

VPHWP, Victorian Public Health and Wellbeing Plan.

## RESULTS

Overall, there has been a decline in indicators from the first VPHWP, excluding the ‘Included other strategies related to oral health (sugar, smoking, diabetes etc.)’ indicator and a slight improvement to the ‘Supported the Achievement Program’ indicator, before it declined again in the VPHWP 2019–2023. There was relatively little change for the ‘Linked healthy eating and oral health’, ‘Included the Smiles 4 Miles program’ and ‘Advocated for fluoridated drinking water’ indicators between the VPHWP 2011–2015 and the VPHWP 2019–2023.

The results of the statistical analysis are presented in Table 2, which indicates the direction of the trends for the inclusion of oral health key terms according to the indicators between the consecutive MPHWP desktop audits. There were statistically unfavourable changes to the ‘Included oral health as a priority’, but statistically favourable changes to the ‘Included other strategies related to oral health (sugar, smoking, diabetes etc.)’ indicator between the VPHWP 2011–2015 and the VPHWP 2015–2019. There was a statistically negative unfavourable change for the ‘Supported the Achievement Program’ indicator between the VPHWP 2015–2019 and the VPHWP 2019–2023. Other changes between the consecutive VPHWPs were not statistically significant.

**Table 2:** Changes in MPHWP indicators between the two consecutive VPHWPs, direction being favourable or unfavourable, and level of statistical significance

MPHWP indicator	∆VPHWP 2011–2015 and the VPHWP 2015–2019 (%)	*p*-value	∆VPHWP 2015–2019 and the VPHWP 2019–2023 (%)	*p*-value	∆VPHWP 2011–2015 and the VPHWP 2019–2023 (%)	*p*-value
Included oral health as a priority	−19	0.002^*^	−6	0.254	−25	<0.001^*^
Linked health eating and oral health	−8	0.125	+1	0.798	−6	0.2354
Supported the Achievement Program	−6	0.254	−14	0.011^*^	−20	0.002^*^
Included the Smiles 4 Miles program	−6	0.133	+1	0.708	−5	0.208
Advocated for fluoridated drinking water	−5	0.103	+1	0.567	−4	0.259
Included other strategies related to oral health (sugar, smoking, diabetes etc.)	+57	<0.001^*^	+3	0.418	+59	<0.001^*^

VPHWP, Victorian Public Health and Wellbeing Plan; + favourable; − unfavourable; ∆ change.

^*^Statistically significant.

## DISCUSSION

A focus on reducing the broader risks factors related to oral health have significantly increased within MPHWPs following the publication of the VPHWP 2015–2019 and the VPHWP 2019–2023. However, our desktop audits identified that explicit prioritization of oral health has largely reduced over time since the VPHWP was published in (2011), although oral health prevention efforts remain captured in the Victorian oral health plans. Our findings indicate that while public health plans have strengthened their focus on the prevention of NCDs, a common risk factor approach can be leveraged by prioritizing oral health due to its significant health and wellbeing, social and economic impacts. We hypothesize that the release of the Victorian oral health plans and consecutive VPHWPs, which did not specify oral health as a priority, reduced the importance of oral health as a focus for local government authorities.

Previous research in rural and remote areas in Victoria has also shown that only 41.6% of the 2017–2021 MPHWPs had some mention of oral health (Dickson‐Swift and Crocombe, 2022). Prioritizing oral health for rural and regional areas is of particular importance because communities generally have poorer oral health, limited access to community water fluoridation and dental services, and may engage in more health risk behaviours such as excess sugar intake and smoking (Victorian Department of Health, 2020; Crocombe *et al.*, 2022).

Although we were unable to identify any empirical research to demonstrate poor oral health precedes overweight and obesity from a life course approach, it is well-known that both are unequally distributed across the socioeconomic spectrum (Moore and Cunningham, 2012; Moysés, 2012), and likely to share antecedent risk factors. We contend there is clear epidemiological logic around the co-benefits for the improvement of population oral health.

Upstream public health interventions such as implementing taxation on SSBs have focussed on preventing overweight and obesity (Sainsbury *et al.*, 2020), but the case could be strengthened by including the oral health impacts. More importantly, there are important considerations in addressing health equity (Lal *et al.*, 2017; Ananthapavan *et al.*, 2020), especially when 39% of Australians have delayed or avoided dental care due to costs (Australian Institute of Health and Welfare, 2023). Although local government authorities cannot impose taxation on SSBs, they can support healthy eating choices and can have an advocacy role to garner public awareness for intervention implementation (Sainsbury *et al.*, 2020).

The umbrella review of public health policies and their impact of health inequalities in high-income countries has shown there is some evidence for their effectiveness depending on the public health field (Thomson *et al.*, 2018). The observed impact of public health policy can take several years, if not decades. For example, grass roots community advocacy led to the inclusion of community water fluoridation within one rural Victorian Shire’s MPHWP in 2015, and the community was able to access fluoridated water in 2021 (Dickson‐Swift et al., 2023). The disappointing findings from our study reveal important implications for prioritizing oral health given dental service provision is largely excluded from Australia’s universal health insurance system, Medicare.

Firstly, there is a need to recognize that the current individualistic clinical paradigm, being that oral health issues are a responsibility of the dental profession, does not reduce health inequities (Watt *et al.*, 2019) because access to oral healthcare for the most part remains a privilege for those who can afford it. Secondly, a concerted effort to explicitly include and reference oral health within a common risk factor approach to NCDs within high-level population health policy documents is critical to strengthen the importance of oral health promotion. Thirdly, a broader understanding between poor oral health and its role in the prevention of NCDs requires significant attention by non-dental experts given the positive impacts that increasing oral health literacy can have to the public and health care providers (Horowitz *et al.*, 2020) and their consequent co-benefits.

Previous reviews have identified the important role of non-dental professionals during pregnancy and early childhood such as medical practitioners, nurses and midwives, and community healthcare workers (George *et al.*, 2019; Riggs *et al.*, 2019; Nation *et al.*, 2022). For example, clinical leadership by the American Academy of Pediatrics has enabled the delivery of fluoride varnish application by non-dental professionals since 2008 (Hanlon, 2010) in the USA under the Medicaid program for young children. Non-invasive cost-effective interventions such as the application of fluoride varnish and silver fluoride (Nguyen et al., 2020, 2022) are opportunities for broader implementation and delivered by non-dental professionals. In Victoria, the same intervention could be implemented through the Victorian Maternal and Child Health Service, which is a responsibility of local government authorities for program delivery.

The scoping review on the policy environment to integrate oral health in primary healthcare makes an important observation, that dental professional associations who assert that the dental profession has overall responsibility for population oral health may not respond to the needs of disadvantaged populations (Harnagea *et al.*, 2017). Therefore, public health practitioners who are closer to the needs of disadvantaged populations should promote the prioritization of oral health through population health policy development and health policy advocacy, wherever possible. Keeping priority for oral health visible such as advocating to drink tap water, preferably fluoridated, including within LGAs that have community water fluoridation serves two purposes: (i) to ensure support for community water fluoridation and (ii) promotes oral health literacy to the general population. It can also mitigate the risk for its cessation as evident in Queensland, when the state government powers were transferred to local government authorities in 2012, resulting in a 4% decrease in population coverage between 2010 and 2017 (Stormon and Lalloo, 2020).

Regulatory considerations would be required to strengthen oral health promotion and the implementation of population oral health interventions. For example, in 2022, the Victorian Department of Health revised the *Drugs, Poisons and Controlled Substances Regulations 2017*, to enable registered Aboriginal and Torres Strait Islander health practitioners to apply fluoride varnish to children aged 3–17 years (Victorian Department of Health, 2022b). The implementation of school dental services targeted to Victorian public schools from 2020 is aligned with all four priorities of the four target goals of the *Victorian Action Plan to Prevent Oral Disease 2020–2030,* to increase the proportion of children entering primary school without dental cavities to 85% and decrease the proportion of Victorian adults with moderate or severe gum disease to 23% (Victorian Department of Health, 2020).

- Priority 1: Improve the oral health of children;
- Priority 2: Promote healthy environments;
- Priority 2: Promote healthy environments;
- Priority 4: Improve oral health promotion, screening, early detection and prevention services.

## LIMITATIONS

To date, it remains unclear how effective the inclusion of oral health within the MPHWPs supports effective implementation of oral health promotion initiatives in Victoria. Internal reporting at DHSV shows the gap between dental caries experience is narrowing for children aged 0–5 years, between Aboriginal and Torres Strait Islander children and non-Indigenous children between 2008/09 and 2020/21 (Dental Health Services Victoria, 2022). During this time, the Healthy Families Healthy Smiles program was implemented in 2012 to train health and early childhood professionals working with young children to promote oral health (Dental Health Services Victoria, 2020b).

The interpretation of the data should be done with caution given it only includes the state of oral health for children who were users of Victorian public dental services and are not representative of the general population. The causal impacts of population health interventions are difficult to establish given the wide array of pre-existing trends and concurrent events. To date, it is unclear what was the driving intervention in reducing the oral health inequities between Aboriginal and Torres Strait Islander children and non-Indigenous children. Future research should consider how public policy impacts on populations at greater risk for oral diseases such and Aboriginal and Torres Strait Islander people and people living in rural and regional areas to ensure there are no adverse effects in widening health inequity.

It is likely there were inconsistencies in how the desktop audits were completed, given they were conducted by different individuals between 2014 and 2022. This may explain the anomaly for a very low proportion of local government authorities included strategies to address common risk factors strategies related to oral health such as reducing sugar intake and reduced smoking rates within the MPHWPs following the VPHWP 2011–2015 publication. In addition, there may be specific health promotion programs that are relevant to oral health but was not captured in the desktop audits such as the Healthy Eating Advisory Service. Some MPHWPs developed by local government authorities were embedded within their LGA plans. The binary interpretation of the oral health key terms may also fail to capture whether there are multiple mentions of oral health within an MPHWP, which could indicate and strengthen priority for oral health in these documents. Finally, it is also unknown whether there is a differential public policy impact when MPHWPs are incorporated into the overarching local government authority plans.

Historically, dental caries has largely been the focus of prevention efforts for oral diseases due to their highest prevalence. The prevalence of periodontitis is significantly less than dental caries but has larger disease burden measured in years lived with disability, which reinforces urgent prevention is required targeting periodontitis (Nguyen *et al.*, 2022). Future analyses that broaden the search terms to include other oral diseases such as periodontal disease and head and neck cancer, and establishing oral health outcomes surveillance systems for LGAs would be important to monitor the impact of VPHWPs and the MPHWPs.

## CONCLUSION

The first VPHWP published in (2011) provided strong direction to prioritize oral health in 2014, but this has not persisted in the 2015 and 2019 plans. Our study demonstrates a multi-sectorial approach is needed to address the burden of NCDs, and that increased efforts to prevent dental caries among children are likely to yield significant co-benefits including overweight and obesity prevention. We emphasize that public health practitioners and health professionals need to play a greater role in improving population oral health.

## Supplementary Material

daad086_suppl_Supplementary_File
