# Supplementary material for: Prioritizing population oral health through public policy in Australia: the Victorian experience
Source: Health Promot Int. 2023 Aug 9;38(4):daad086. doi: 10.1093/heapro/daad086 (PMC10411047; doi:10.1093/heapro/daad086)
Supplement: daad086_suppl_Supplementary_File [file daad086_suppl_supplementary_file.pdf]

Oral health and Municipal Public Health and Wellbeing Plans 2021-2025

Audit Report 2022

Promoting oral health and preventing oral disease in the community

The *Victorian Public Health and Wellbeing Plan 2019–2023* (VPHWP) continues the Victorian Government's vision for the public health and wellbeing of Victorians: '*A Victoria free of the avoidable burden of disease and injury so that all Victorians can enjoy the highest attainable standards of health, wellbeing and participation at every age.'*

Municipal Public Health and Wellbeing Plan (MPHWP) 2021-2025

The *Victorian Public Health and Wellbeing Act* (2008) recognises the key role of Councils in improving the health and wellbeing of people in their municipality. Section 26 of the Act requires each Council to prepare a Municipal public health and wellbeing plan every four years, within 12 months of a Council general election.

The VPHWP emphasises the role of local government in supporting healthy communities and recognises the MPHWP as a key strategic planning mechanism for public health and wellbeing efforts at the community level.

Local government area profiles

The Department of Health (the Department) in partnership with DHSV produces statistical profiles of geographic areas that provide a broad range of data about each geographic area including indicators relating to population composition and growth, diversity, socio-economic status, community strength, health status and wellbeing, and service performance and utilisation.

In August 2020, Dental Health Services Victoria (DHSV) distributed oral health profiles to individual local governments with data on health-related behaviours that have an impact on oral health. The profiles also contained data on preventable hospital admissions for dental conditions for children aged 0-4 years. The information was provided to the councils to assist in oral health promotion efforts in their communities and inform the development of the MPHWP.

34 per cent of councils in Victoria included

oral health as a priority in their

Municipal Public Health and Wellbeing Plan

2021-2025

Previously, DHSV conducted a desktop audit of the MPHWP 2013-2017 and shared these findings to stakeholders. In May 2022, DHSV performed a desktop audit of the MPHWP 2021-2025 and made comparisons between the two reviews on the content analysis relevant to oral health. Overall, there were no significant changes for LGAs including oral health in the review except there was a significant reduction of LGAs explicitly stated they support the Achievement Program. The desktop audit was supported by the Department.

Oral health - a priority

There are 79 local government areas (LGAs) in the state of Victoria. They are constituted as cities, shires, rural cities and, in one case, a borough. Preliminary findings show, out of the **79** LGAs, **27 (34%)** LGAs included oral health as a priority and **52 (66%)** LGAs did not include oral health as a priority in their MPHWPs 2021-2025.

Of the **27** LGAs that included oral health as a priority:
- **10 (33%)** LGAs linked oral health with healthy eating strategies,
- **4 (15%)** LGAs included supporting the Achievement Program,
- **4 (15%)** LGAs included implementation of the Smiles4Miles Program, and
- **2 (7%)** LGAs included advocating and promoting fluoridated drinking water.

Of the **79** LGAs, **76 (96%)** LGAs included other strategies related to oral health:
- **28 (35%)** Reduction of sugar intake
- **39 (49%)** Tobacco control
- **26 (33%)** Preventing diabetes
- **27 (34%)** Preventing obesity.

Audit of the Municipal Public Health and Wellbeing Plan 2021-2025

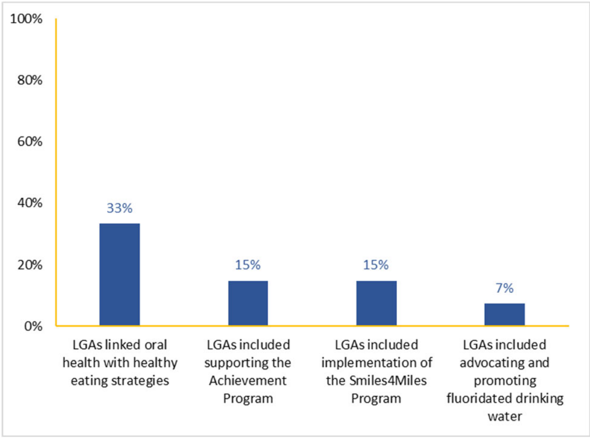

Examples of how oral health was included in the MPHWP 2021-2025:
- Advocate to improve breastfeeding rates
- Increased fruit and vegetable consumption
- Increase consumption of water
- Decrease daily consumption of sugar sweetened beverages
- Support the Victorian Achievement Program (VAP): Healthy Eating & Oral Health
- Investigate use of the Smiles 4 Miles program for kindergartens
- Deliver Smiles 4 Miles: three core messages: drink well, eat well and clean well
- Support advocacy for fluoridation of local drinking water supply (town and tank water)
- Reducing tobacco-related harm
- Decrease harmful alcohol and drug use
